# Cytotoxic effects of vincristine on tumour subpopulations separated from pulmonary nodules.

**DOI:** 10.1038/bjc.1983.183

**Published:** 1983-08

**Authors:** D. J. Grdina, R. A. White, J. J. Stragand

## Abstract

**Images:**


*Br. J. Cancer* (1983), **48**, 279–287

### Cytotoxic effects of vincristine on tumour subpopulations separated from pulmonary nodules

D. J. Grdina[1], R.A. White [2], & J.J. Stragand[3]

[1]*Department of Experimental Radiotherapy,* [2]*Department of Biomathematics, and* [3]*Department of Laboratory Medicine The University of Texas M.D. Anderson Hospital and Tumour Institute Houston, Texas 77030, U.S.A.*

**Summary** The cytotoxic and stathomokinetic effects of vincristine (VCR) on murine fibrosarcoma (FSa) cells, grown either *in vitro* as primary cultures or *in vivo* as micro- or macroscopic pulmonary nodules, were determined and compared. FSa cells were separated and synchronized on the basis of size by centrifugal elutriation. Flow microfluorometry (FMF) was used to determine the cell-cycle parameters and the relative synchrony of the separated populations, thus allowing determination of age-dependent cytotoxicity. The colony-forming efficiencies (CFE) of these cells were determined using a lung colony assay. Synchronized cell population of FSa cells, separated by centrifugal elutriation, were injected into recipient animals and exposed 20 min later to a single dose of VCR to determine their age-specific sensitivity. Under these conditions there appeared to be a suggestion of an enhanced killing of cells enriched in the $G_2+M$ phase. However, following prolonged VCR exposure *in vitro* (e.g., $2.5\,\mu g\,ml^{-1}$; 25 ml for 24 h) to primary cultures of FSa cells or *in vivo* (e.g., $0.25\,mg\,kg^{-1}$ per fraction i.p.; 5 fractions in 24 h) to macroscopic pulmonary tumour nodules, elutriated FSa cell populations most enriched with $G_1$ phase cells exhibited the lowest CFE. If under either condition exposed cells were allowed to recover in the absence of VCR for 24 h prior to their removal and separation, FSa cell survival increased in each of the elutriated populations. In contrast, while $G_1$ enriched cell populations from *in vitro* exposed cultures still exhibited a significant reduction in CFE, no such age-specific response was observed for *in vivo* exposed macroscopic pulmonary nodules. The stathomokinetic effect of VCR on FSa cells was also readily observed *in vitro* using FMF analysis (e.g., an increase of from 20 to 38% in the $G_2+M$ phase compartment). While no such effect was observed *in vivo* using FMF analysis, cluster of mitotic figures were observed. The mitotic indices (MI) of the *in vivo* exposed FSa cells increased from 2.5 to 8.4%.

The principal mode of action of vincristine (VCR), a member of the vinca alkaloids, at low concentrations is through interference with microtubulin polymerization and mitotic spindle formation in mammalian cells, leading to cell arrest in mitosis (Himes *et al.*, 1976; Owellen *et al.*, 1974). The ability of VCR to arrest cells in metaphase, however, does not always correlate with phase-specific cytotoxic activity (Camplejohn, 1980). Variations in the age response of mammalian cells to VCR have been reported for several cell lines including $G_1$ phase sensitivity for P388 leukemia cells (Hill & Whelan, 1981), S phase sensitivity for hamster NIL8 cells (Csuka *et al.*, 1980), and late S and $G_2$ phase sensitivity for human 3025 cells (Wibe, 1980).

In the characterization of the phase-specific cytotoxicity of antitumour agents such as VCR, the relative cell-stage responses are routinely assayed under *in vitro* conditions that do not simulate the complex conditions existing *in vivo*. It is advantageous, therefore, to be able to evaluate the phase-specific cytotoxic effectiveness of chemotherapeutic agents such as VCR on selected target cells *in vivo*, as well as *in vitro*.

In this communication we describe the cytotoxic effectiveness of VCR on murine FSa cells following prolonged exposure under *in vitro* or *in vivo* conditions. The design of the experimental protocol is presented in Figure 1 and is based on techniques described elsewhere (Grdina *et al.*, 1979; 1980; Grdina, 1982) to characterize phase-specific antitumour agents under both *in vitro* and *in vivo* conditions.

### Materials and methods

### *Mice and tumour*

Female $C_3Hf/Kam$ mice, 10–12 weeks old, from our specific pathogen-free breeding colony, and a methylcholanthrene-induced fibrosarcoma were used in these experiments (Suit & Suchato, 1967). Tumours, sixth generation isotransplants, were made into viable cell suspensions and either cultured *in vitro* (Grdina *et al.*, 1978*a*) or injected into untreated recipient mice to give rise in 13 days to 100–150 visible pulmonary nodules (Grdina *et al.*, 1978*b*).

Correspondence: D. J. Grdina.
Received 19 January 1983; accepted 4 May 1983.

© The Macmillan Press Ltd 1983.

*Tumour cell suspension*

Single cell suspensions were prepared from pulmonary tissue and tumour nodules according to a method described in detail elsewhere (Grdina, 1982). Since no advantage has been observed following the tedious excision of each individual tumour nodule from the surrounding lung tissue, entire tumour-bearing lungs were minced and digested with trypsin (Grdina, 1982). Following this procedure, lung tissue floated to the surface and the highly enriched but undigested tumour tissue settled to the bottom of the beaker. Viable tumour cells in single-cell suspension were collected in the upper two-thirds of the suspension mixture. Cell viability determined by phase-contrast microscopy was routinely >95%. The yield of viable tumour cells was about $8.5 \times 10^7 g^{-1}$ tissue (Grdina & Hunter, 1982).

*Culture conditions*

FSa tumour cells were cultured *in vitro* under conditions described in detail elsewhere (Grdina *et al.*, 1982*a*). Viable cells $1.5 \times 10^7$, were seeded into 32-ounce glass culture bottles, 20 bottles per experiment, and incubated at 37°C in a water-saturated atmosphere of 5% $CO_2$ and air. The growth medium was a modified McCoy's 5A medium supplemented with 20% foetal calf serum (Humphrey *et al.*, 1970). After a 24 h incubation, the supernatant containing floating cells was discarded and 20 ml of fresh medium was added to each culture bottle. The attached tumour cells were then incubated for an additional 24 h prior to drug exposure.

*Cell separation* (see Figure 1)

FSa cells derived directly from pulmonary nodules or following growth *in vitro* were separated by centrifugal elutriation (Grdina *et al.*, 1978*a*; Grdina, 1982). Elutriation was performed using a Beckman JE-6 elutriator rotor and a standard Beckman chamber. This system and all of the associated tubing were sterilized with 70% ethanol and maintained at 4°C. The separation medium was a modified McCoy's 5A supplemented with 5% foetal calf serum containing DNase (Deoxyribonuclease 1; Sigma Chemical Co., St. Louis, MO) at a final concentration of $0.1\,mg\,ml^{-1}$ and 5 mM 2-naphthol 6–8 disulphonic acid to reduce cell clumping (Shortman, 1973). With a rotor speed of $1525\,rev\,min^{-1}$, between $2 \times 10^8$ and $3 \times 10^8$ cells, suspended in 20 ml of medium were introduced into the elutriator chamber of a flow rate of $5.4\,ml\,min^{-1}$. The rotor speed was held constant throughout the separation, and the flow rates were varied by equal increments from 5.4 to $27.4\,ml\,min^{-1}$. Twelve 50 ml fractions (F) were collected and stored at 4°C. Cells collected in each fraction were counted by haemacytometer and by Coulter counter (model ZBI, Coulter Electronics, Hialeah, FL). F1, containing small cells and cellular debris, and F12, containing a heterogeneous mixture of cells, were discarded.

**Figure 1** Experimental protocols describing *in vitro* and *in vivo* experiments performed to characterize the effectiveness of VCR on FSa tumour cells.

*Flow microfluorometry*

The DNA content of individual cells in suspensions was measured by flow microfluorometry (FMF) using an ICPII flow cytometer (Phywe Co., Gottigen, Germany). Cells were fixed in 70% ethanol and stained with $50\,mg\,ml^{-1}$ mithramycin (Mithracin, Chas. Pfizer and Co., Inc., New York, NY) in a solution containing 7.5mM $MgCl_2$ and 12.5% aqueous ethanol (Grdina *et al.*, 1982*a*). the resultant histograms of DNA fluorescence were computer analyzed (Johnston *et al.*, 1978). Since FSa cells are heteroploid (i.e., 60–70 chromosomes) and contain about 1.8 times as much DNA as normal diploid cells, an estimate of the normal cell contamination in each of the tumour-cell suspensions was made by determining the area under the $G_1$ normal and dividing it by the area under the entire FMF histogram (Grdina, 1982; Grdina & Hunter, 1982). All cell counts used for the clonogenicity assays were adjusted to represent only tumour-cell numbers.

*Mitotic index*

The ability of VCR to arrest tumour cells in mitosis following prolonged *in vivo* exposure was determined. Five fractions, $0.25\,mg\,kg^{-1}$ each, of VCR administered i.p. at 6h intervals. Mitotic figures were scored in 3 mice per group at 3, 4, 18, and 25h following the last injection. Following sacrifice and excision, tumour-bearing lungs were fixed in neutral buffered formalin for 48h. Five-$\mu$m histological sections were prepared, mounted, and stained. Five to 10 tumour nodules per mouse, in which the vascular supply was clearly visible, were selected. At 1000× magnification, 5 microscope fields adjacent to and surrounding the vascular surface were examined for the number of mitotic figures per total number of cells. Mitotic figures were determined by counting a minimum of 1500 cells per experimental point.

*VCR exposure* in vitro

Stock solutions of vincristine sulfate (Oncovin), obtained from the Eli Lilly Co., Indianapolis, IN, were made up in bacteriostatic sodium chloride solution at a concentration of $0.1\,mg\,ml^{-1}$.

FSa cells derived from pulmonary nodules were grown *in vitro* for 48h prior to exposure to drug. At that time, the media was changed and the attached cells were exposed to a $2.5\,\mu g\,ml^{-1}$ concentration of VCR (25ml/bottle) for either 4 or 24h. Following exposure, the cells were washed free of VCR and either immediately harvested and separated by centrifugal elutriation or allowed to remain in culture for an additional 24h before harvesting and separation.

*VCR exposure* in vivo

Two experimental protocols were used to evaluate the effectiveness of VCR *in vivo*. In all studies at least two reproducible experiments were performed. The effectiveness of a single dose of $1\,mg\,kg^{-1}$ VCR was evaluated on FSa cells recently trapped in the lungs of test animals. Prior to drug treatment, FSa cells grown as pulmonary nodules were harvested, made into single-cell suspensions, and separated by centrifugal elutriation into subpopulations of cells enriched in $G_1$, S, or $G_2+M$ phase (Grdina *et al.*, 1979; 1980; Grdina, 1982). Known numbers of viable cells from each of these fractions were then injected i.v. into corresponding groups of recipient animals, 20 mice per group. Twenty minutes after injection of the tumour cells, 10 mice in each group were injected i.p. with VCR. Thirteen days later the animals were sacrificed, and the resulting colonies in control and treated groups were counted and compared (Grdina *et al.*, 1979; 1980; Grdina, 1982).

The effect of multiple doses of VCR on cell killing was determined using mice bearing 13-day-old pulmonary nodules. Thirty experimental animals having from 100–150 pulmonary nodules were injected i.p. with 5 fractions of VCR at a dose of $0.25\,mg\,kg^{-1}$ per fraction. Injections were made at 6h intervals. Either 1 or 24h after the last injection, animals were sacrificed and their lungs were removed. Suspensions of FSa cells were made and separated by centrifugal elutriation. The CFE of these cells was determined and compared to corresponding untreated controls using a lung colony assay (Grdina, 1982; Grdina & Hunter, 1982).

*Lung colony assay*

The CFE of FSa cells was determined in a lung colony assay. Recipient mice, with their hind legs shielded, were whole-body irradiated with 10Gy 24h before use. These mice were then injected with known numbers of viable FSa cells, corrected for normal cell contamination from each of the elutriator fractions and the unseparated control cell (USC) population. Each aliquot of cells also contained $2\times10^6$ heavily irradiated (HIR) (100Gy) FSa cells. HIR cells were not separated by centrifugal elutriation. Thirteen days after the mice were killed, their lungs were removed, the lobes separated and fixed in Bouin's solution, and tumour colonies counted.

### Results

The recovery of cells following centrifugal elutriation was routinely >90%, and the viability

**Figure 2** Percentage of FSa tumour cells, derived from pulmonary metastases, distributed among the various cell-cycle phases plotted as a function of elutriator fraction number. (○) G; (△) S; (□) $G_2$+m.

of these cells, determined by phase contrast microscopy, was >95%. Using a lung colony assay, the CFE of untreated cells collected from each of the elutriator fractions ranged from 1–3%. Surviving fractions were determined by comparing the CFE's of untreated cells from each of the elutriator fractions with those of corresponding treated groups.

Presented for comparison in Figure 2 are representative data describing the percentages of tumour cells in $G_1$, S, and $G_2$+M phase, as determined by FMF analysis, in each of the elutriator fractions. In general, $G_1$, S, and $G_2$+M cells were most enriched in fractions 2 through 4, 5 through 7, and 8 through 11, respectively. This type of analysis was routinely performed following each elutriation of cells. No difference was observed in the relative cell cycle distribution of cells as a function of elutriator fraction number between control and treated cell populations.

To determine the possible magnitude of the stathmokinetic effects of VCR on FSa cells, tumour cells were exposed under *in vitro* conditions to a dose of 6.25 mg of VCR for 24 h. Presented in Figure 3 for comparison are representative FMF profiles describing the DNA contents of control and drug-treated cells. Under these conditions, VCR gave rise to a 2-fold build-up of cells having $G_2$+M phase DNA contents.

To determine the phase specificity, if any, of VCR, a single dose was administered 20 min

**Figure 3** Representative FMF profiles of unseparated FSa cell populations *in vitro*. (a) unexposed FSa control population; $G_1$(N) refers to normal diploid lung cells in $G_1$ (17%); $G_1$(T) refers to tumour cells in $G_1$ (59%); $G_2$+M(T) refers to tumour cells in $G_2$+M (20%) (b) FSa cells exposed to VCR for 24 h; $G_1$(N) (16%), $G_1$(T) (25%), and $G_2$+M(T) (38%); coefficients of variation of $G_1$ tumour peak untreated 4%, treated 5%.

following the injection and entrapment in the lungs of elutriated FSa cells. Under these conditions, VCR exhibited little, if any, cytotoxic effect (Figure 4). The USC population exhibited a surviving fraction of 92%. However, cell populations enriched in $G_2+M$ phase by elutriation (e.g., fractions 8–11) consistently showed a slightly reduced survival when assayed in this manner in 3 separate experiments, suggesting a possible sensitivity for these cells.

**Figure 4** Percent surviving fraction of FSa cells to a single dose of VCR elutriator fraction number. Twenty min following the i.v. imjection of tumour cells, $1.0\,mg\,kg^{-1}$ of VCR was injected i.p. Error bars represent 1 of the mean.

The cytotoxic effectiveness of VCR following prolonged exposure was also determined. FSa cells were exposed first *in vitro* to the same concentration of drug, but the exposure times were either 4 or 24 h. The ability of the treated population to recover following VCR exposure was also determined. Representative data are presented in Figure 5.

Following exposure for 4 h, elutriator fractions most enriched in S phase cells appeared to be the most resistant (Figure 5). USC exhibited a surviving fraction of 30%. Exposure for 24 h reduced cell survival of the USC population to 2.5%. The greatest cell killing, however, was observed in elutriator populations enriched in $G_1$ phase cells.

Cell survival was enhanced in the USC population (4.5% surviving fraction) and FSa subpopulations collected in elutriator fractions 4–11 if VCR-exposed cells were incubated in drug-free medium for 24 h prior to elutriation. Notable exceptions (i.e., no improvement in cell survival) were observed for $G_1$-enriched FSa cells collected in fractions 2 and 3.

**Figure 5** Percent surviving fraction as a function of elutriator fraction number of FSa cells following exposure to VCR ($2.5\,\mu g\,ml^{-1}$; 25 ml per bottle) *in vitro* for 4 h/0 h (cells harvested immediately after exposure) (●), 24 h/0 h (cells harvested immediately) (▲), and 24 h/24 h (cells harvested 24 h following exposure) (○). Following harvesting, FSa cells were separated by centrifugal elutriation and CFE were determined using a lung colony assay. Error bars represent 1 of the mean.

Following the characterization of the stathmokinetic and cytotoxic effectiveness of VCR against FSa cells *in vitro*, experiments were designed to study the *in vivo* effectiveness of this agent. To ensure a sufficient tumour mass for study, FSa cells were grown *in vivo* for 13 days to form macropulmonary nodules. Because of the large tumour burden, and to approximate the exposure times *in vitro*, VCR was administered to tumour-bearing animals in five fractions ($0.25\,mg\,kg^{-1}$ per fraction) over a 24 h period. Presented for comparison in Figure 6 are representative FMF profiles describing the DNA contents of FSa cells derived from 13 day old pulmonary nodules that were either untreated or treated. In contrast to the data presented in Figure 3, no change in DNA distribution was observed following VCR exposure *in vivo*.

**Figure 6** Representative FMF profiles of unseparated FSa cell populations *in vivo*. (a) unexposed control FSa cells; $G_1$(N) (35%), $G_1$(T) (59%), and $G_2$+M(T) (15%). (b) FSa cells from tumour nodules exposed to 5 F of VCR (0.25 mg $kg^{-1}$ per fraction) over a 24 h period; $G_1$(N) (32%), $G_1$(T) (61%), and $G_2$+M(T) (14%). Coefficients of variation of $G_1$ tumour peak untreated 6%, treated 5%. MI refers to mitotic index.

**Figure 7** Mean mitotic index of FSa cells exposed as 13-day-old pulmonary tumour nodules as a function of time following 5 fractions (0.25 mg $kg^{-1}$ per fraction) of VCR administered over a 24 h period. Error bars represent 1 of the mean.

Because of the inability to demonstrate a stathmokinetic effect of VCR using FMF, experiments were designed to evaluate the effectivenss of this agent by measuring changes in mitotic indices (MI) following exposure. Five fractions of VCR (0.25 mg $kg^{-1}$ per fraction) were administered over a 24 h period to repeat the studies described earlier using FMF analysis. Under these conditions, the mean MI of these cells increased from $\sim$2 to 8 (Figure 7). The MI then decreased as a function of time following the last injection to values below that of control by 18 h.

To facilitate a comparison of the cytotoxic effects of VCR on FSa cells following prolonged exposure under both *in vitro* and *in vivo* conditions, VCR was administered to tumour-bearing mice in 5 fractions over a 24 h period. If animals were sacrificed 1 h following the last injection, cell populations most enriched with $G_1$ phase cells exhibited the lowest surviving fractions. The USC population had a surviving fraction of 42%. The reduced clonogenicity of $G_1$ enriched FSa populations was not observed, however, if animals were allowed to survive an additional 24 h after treatment. In contrast to the *in vitro* survival data presented in Figure 5, elutriator fractions enriched in $G_1$ phase cells contained approximately the same proportion of surviving cells as contained in the remaining elutriator fractions (Figure 8) and the USC population (surviving fraction of 68%).

### Discussion

In earlier reports we described in detail procedures by which chemotherapeutic agents could be characterized *in vivo* with respect to phase specificity in cell killing (Grdina *et al.*, 1979; 1980). Our results using hydroxyurea, adriamycin, cytosine arabinoside, bleomycin, and *cis*-diammindichloroplatinum were found to compare favorably with those obtained under *in vitro* conditions (Meyn *et al.*, 1980).

Subsequent to these studies we characterized the age response of FSa cells to ionizing radiation under *in vitro* and *in vivo* conditions, the latter either as single cells trapped in the lungs of mice or as 14 day old pulmonary tumour nodules (Hunter *et al.*, 1979; Grdina & Hunter, 1982). Again, there was excellent agreement in the qualitative age response exhibited under either *in vitro* or *in vivo*

**Figure 8** Percent surviving fraction as a function of elutriator fraction number of FSa cells following prolonged *in vivo* exposure to VCR. Five fractions (f) of VCR at a dose of 0.25 mg kg$^{-1}$ were injected i.p. Each dose was separated by 6 h, and tumour-bearing animals were sacrificed at 1 h (○) or 24 h (▲) following the last injection. Tumours were then excised, made into single cell suspensions, and cells were separated by centrifugal elutriation. CFE were determined using a lung colony assay. Error bars represent 1 of the mean.

conditions. These results support the efficacy of our *in vivo* procedure to characterize the phase specific cytotoxicity of selected chemotherapeutic agents *in vivo*. We have applied this procedure to study not only the effect of a single dose of VCR on tumour cells *in vivo,* but also the cytotoxic and stathmokinetic effects of either multiple exposures *in vivo* or prolonged exposure *in vitro*.

FSa cells, derived from artificially induced pulmonary tumour nodules, were effectively separated by the method of centrifugal elutriation into subpopulations enriched in $G_1$, S, and $G_2$+M phase cells. To ensure that potential changes in cell size without concomitant changes in DNA content of treated cells would be monitored, FMF analysis was performed on cells from each elutriation fraction following separation. Under the conditions described, the response of FSa cells to VCR was unchanged regardless of whether exposure was before or after separation by centrifugal elutriation. Similar results with respect to the lack of an effect exerted by the method of centrifugal elutriation on the age response of separated cells has been reported by others (Keng & Wheeler, 1980; Grdina & Hunter, 1982).

No discernible difference with respect to either cell yield following the preparation of single-cell suspensions or sedimentation profiles following the separation of cells by centrifugal elutriation was observed between cell populations derived from VCR-treated or untreated control FSa pulmonary tumours.

The stathmokinetic effect of VCR on FSa cells, as determined by FMF analysis, was mostly readily demonstrable under *in vitro* conditions. In contrast, little or no effect could be observed using FMF following exposure of FSa cells by VCR *in vivo*. For this reason, MI were also determined for both normal and malignant tissues. It was difficult to determine the MI of FSa lung nodules because large areas in the tumour cross sections contained either few or no mitotic figures or large clusters of mitotic cells, suggesting a non-unifrom exposure to drug. MI determinations were performed, therefore, using capillaries in the tumour cross sections as points of reference. The failure to observe a stathmokinetic effect of VCR *in vivo* using FMF was most probably due, therefore, to the large background of unaffected or underexposed FSa cells. The clustering of mitotic figures is most probably indicative of the non-uniform distribution of VCR.

An attempt was made to characterize the *in vivo* phase cytotoxicity of VCR on FSa cells lodged in the lungs of mice. VCR administered as a single dose *in vivo* was slightly more effective on FSa cells injected from fractions 8–11 that from the other elutriator fractions 2 to 5. However, because of the relative small, albeit consistent, reduction in absolute survival in these fractions, it is not clear whether this apparent relatively weak $G_2$+M phase-specific response is significant or not.

Following prolonged exposure *in vitro* to VCR, however, $G_1$-enriched FSa cells collected in elutriator fractions 2 and 3 exhibited the lowest surviving fractions. The reduced CFE of cells in these fractions after even a short exposure time of 4 h suggests that VCR may have been directly effective against cells in $G_1$. It is probable, however, that some cells damaged in late $G_2$ and M phase survived the division process and progressed to $G_1$ phase at the time of elutriation. This possibility may also explain the failure of cells in fractions 2 and 3 to increase in CFE after 24 h of drug-free incubation following a 24 h exposure, as compared to FSa cells elutriated immediately after exposure.

Cell progression along with other factors must be considered in interpreting the survival data of FSa cells exposed over a 24 h period *in vivo*. During this period, the mitotic index in the FSa lung nodules increased from 1.2–5.5% as a result of the stathmokinetic effect of VCR. In good agreement with the *in vitro* studies, the greatest reduction in CFE was observed in $G_1$-enriched FSa cells collected in elutriator fractions 2 and 3 if animals were sacrificed 1 h following the last exposure. These data again suggest that some damaged cells progressed through the mitotic block and accumulated in $G_1$ at the time the animals were sacrificed. These data do not preclude the possibility that some VCR-arrested cells underwent endoreduplication and became multinucleate cells (Alabaster & Cassidy, 1978; Hartenstein *et al.*, 1973). Cells such as these would, however, be relatively large and would, therefore, have been collected in elutriator fractions 10–12. These cells would not have DNA contents characteristic of $G_1$ phase cells.

The major difference between the cell survival data acquired *in vitro* and *in vivo* was observed for VCR-treated animals that were allowed to survive an additional 24 h following exposure. Under *in vivo* conditions little or no difference in surviving fraction was apparent between the elutriated populations. Specifically, populations enriched in $G_1$ phase cells exhibited no selective reduction in CFE. The failure to exhibit $G_1$ sensitivity under this *in vivo* condition may be due to several factors. VCR-damaged cells *in vivo* may be selectively removed by host surveillance mechanisms. Since elutriation occurs 24 h following treatment, sufficient time would be available for the selective removal of damaged cells. Secondly $G_0$-like nonproliferating FSa cells may be recruited following VCR exposure. These cells, presumably undamaged, could then enter into the progression cycle and, in a manner, dilute the number of VCR-damaged clonogens present 24 h after treatment. Unfortunately, these quiescent cells cannot be readily identified in the FSa tumour system by two parameter FCM methods (Brock *et al.*, 1982). Their presence is strongly suggested, however, from DNA precursor labelling data presented elsewhere (Sigdestad & Grdina, 1981; Brock *et al.*, 1982). Thirdly, repair of VCR-induced damage may be more efficient under *in vivo* as compared to *in vitro* conditions. In particular, a selected cohort of cells in the pulmonary tumours might, in contrast to cells growing *in vitro,* be capable of repairing chemically-produced potentially lethal damage. Any or all of these factors could lead to the observed reduced cytotoxic effect of VCR on $G_1$ enriched populations *in vivo*.

In conclusion, we have characterized the response of FSa cells to VCR grown either as *in vitro* monolayer cultures or as *in vivo* pulmonary tumour nodules. The response of these cells to prolonged exposure to VCR was comparable under both conditions only if elutriation and CFE were determined immediately following prolonged treatment. Cell populations recovered following elutriation, which were enriched in $G_1$ phase cells, exhibited the lower CFE. This may have been due to both direct killing of $G_1$ phase cells, and the progression of damaged $G_2+M$ phase cells into the $G_1$ compartment prior to elutriation. If cells were allowed to recover for 24 h following treatment, CFE was increased for S and $G_2+M$ phase cells under both *in vivo* and *in vitro* conditions. Only populations enriched in $G_1$ phase cells responded differently. These data demonstrate that *in vitro*-devised experiments may not always reflect the *in vivo* kinetic and clonogenic response of target cells to chemotherapeutic agents such as VCR. Through the use of centrifugal elutriation and artifical metastases systems, such as single cells lodged in the lungs (i.e., micrometastases) or 13 day old pulmonary nodules (i.e., macrometastases), the therapeutic effectiveness of selected modalities, either alone or in combination, can routinely and rapidly evaluated.

This work was conducted with the excellent technical assistance of Sandra Jones, Nancy Hunter, and Gary Zin. We are grateful to Debra Palmatary and her staff for the supply and care of the animals used in these experiments.

Animals used in this study were maintained in facilities approved by the American Association for Accreditation of Laboratory Animal Care and in accordance with current United States Department of Agriculture, Department of Health and Human Services, National Institute of Health regulations and standards.

This investigation was supported in part by research grant CA-18628, CA-23270, CA-11430, and CA-06294, awarded by the National Cancer Institute, Department of Health and Human Services.

### References

ALABASTER, O. & CASSIDY, M. (1978). Flow microfluorometric analysis of P388 murine leukemia after administration of vincristine and maytansine *in vivo. J. Natl Cancer Inst.*, **60,** 649.

BROCK, W.A., SWARTZENDRUBER, D.E. & GRDINA, D.J. (1982). Kinetic heterogeneity in density-separated murine fibrosarcoma subpopulations. *Cancer Res.*, **42,** 4999.

CAMPLEJOHN, R.S. (1980). A critical review of the use of Vincristine (VCR) as a tumour cell synchronizing agent in cancer therapy. *Cell Tissue Kinet.*, **13,** 327.

CSUKA, O., SUGAR, J., PALYI, I. & SOMFAI-RELLE, S. (1980). The mode of action of vinca alkaloids. *Oncology*, **37,** 83.

GRDINA, D.J., PETERS, L.J., JONES, S. & CHAN, E. (1978*a*). Separation of cells from a murine fibrosarcoma on the basis of size. I. Relationship between cell size and age as modified by growth *in vivo* or *in vitro*. *J. Natl Cancer Inst.*, **61**, 209.

GRDINA, D.J., PETERS, L.J., JONES, S. & CHAN, E. (1978*b*). Separation of cells from a murine fibrosarcoma on the basis of size. II. Differential effects of cell size and age on lung retention and colony formation in normal and preconditioned mice. *J. Natl Cancer Inst.*, **61**, 215.

GRDINA, D.J., SIGDESTAD, C.P. & PETERS, L.J. (1979). Phase-specific cytotoxicity *in vivo* of hydroxyurea on murine fibrosarcoma cells synchronized by centrifugal elutriation. *Br. J. Cancer*, **39**, 152.

GRDINA, D.J., SIGDESTAD, C.P. & PETERS, L.J. (1980). Cytotoxic effect *in vivo* of selected chemotherapeutic agents on synchronized murine fibrosarcoma cells. *Br. J. Cancer*, **42**, 677.

GRDINA, D.J. (1982). Phase-specific cytotoxicity *in vivo* of hydroxyurea on murine fibrosarcoma pulmonary nodules. *Br. J. Cancer*, **45**, 438.

GRDINA, D.J. & HUNTER, N. (1982). Cyclic-radiation response of murine fibrosarcoma cells grown as pulmonary nodules. *Int. J. Radiat. Oncol. Biol. Phys.*, **8**, 1727.

HARTENSTEIN, R., EHRHART, H. & HOFFMANN, K. (1973). Zellkinetische untersuchungen beim Ehrlich-Ascites tumor unter Vincristine. *Z. Krebsforch*, **79**, 213.

HILL, B.T. & WHELAN, R.D. (1981). Comparative cell killing and kinetic effects of Vincristine or Vindesine in mammalian cell lines. *J. Natl Cancer Inst.*, **67**, 437.

HIMES, R.H., KERSEY, R.N., HELLER-BETTINGER, I. & SAMSON, F.E. (1976). Action of the vinca alkaloids Vincristine, Vinblastine, and Desacetyl Vinblastine amide on microtubules *in vitro*. *Cancer Res.*, **36**, 3798.

HUMPHREY, R., STEWARD, D. & SEDITA, B. (1970). DNA strand scission and rejoining in mammalian cells. *In Genetic Concepts and Neoplasia*, Baltimore: Williams and Wilkins Co. p. 570.

HUNTER, N., PETERS, L.J., GRDINA, D.J., WHITE, R.A. & BARTEL, A. (1979). Radiation sensitivity of murine fibrosarcoma cells separated by centrifugal elutriation. *Radiat. Res.*, **80**, 389.

JOHNSTON, D.A., WHITE, R.A. & BARLOGIE, B. (1978). Automatic processing and interpretation of DNA distributions: Comparisons of several techniques. *Comp. Biomed. Res.*, **11**, 393.

KENG, P.C., & WHEELER, K.T. (1980). Radiation response of synchronized 9L rat brain cells separated by centrifugal elutriation. *Radiat. Res.*, **83**, 633.

MEYN, R.E., MEISTRICH, M.L., & WHITE, R.A. (1980). Cycle-dependent anti-cancer drug cytotoxicity in mammalian cells synchronized by centrifugal elutriation. *J. Natl Cancer Inst.*, **64**, 1215.

OWELLEN, R.J., DONIGIAN, D.W., HARTKE, C.A., DICKERSON, R.M. & KUHAR, M.J. (1974). The binding of Vinblastine to tubulin and to particulate fractions of mammalian brain. *Cancer Res.*, **34**, 3180.

SHORTMAN, K. (1973). Physical procedures for the separation of animal cells. *Ann. Rev. Biophys. Bioeng.*, **7**, 93.

SIGDESTAD, C.P., & GRDINA, D.J. (1981). Density centrifugation of murine fibrosarcoma cells following *in situ* labelling with tritiated thymicine. *Cell Tissue Kinet.*, **14**, 589.

SUIT, H., & SUCHATO, D. (1967). Hyperbaric oxygen and radiotherapy of fibrosarcoma and squamous cell carcinoma. *Radiology*, **89**, 713.

WIBE, E. (1980). Age-dependent cell inactivation of Vincristine alone or in combination with I-Propargyl-5-chloropyrimidin-2-one. *Cancer Res.*, **40**, 2069.


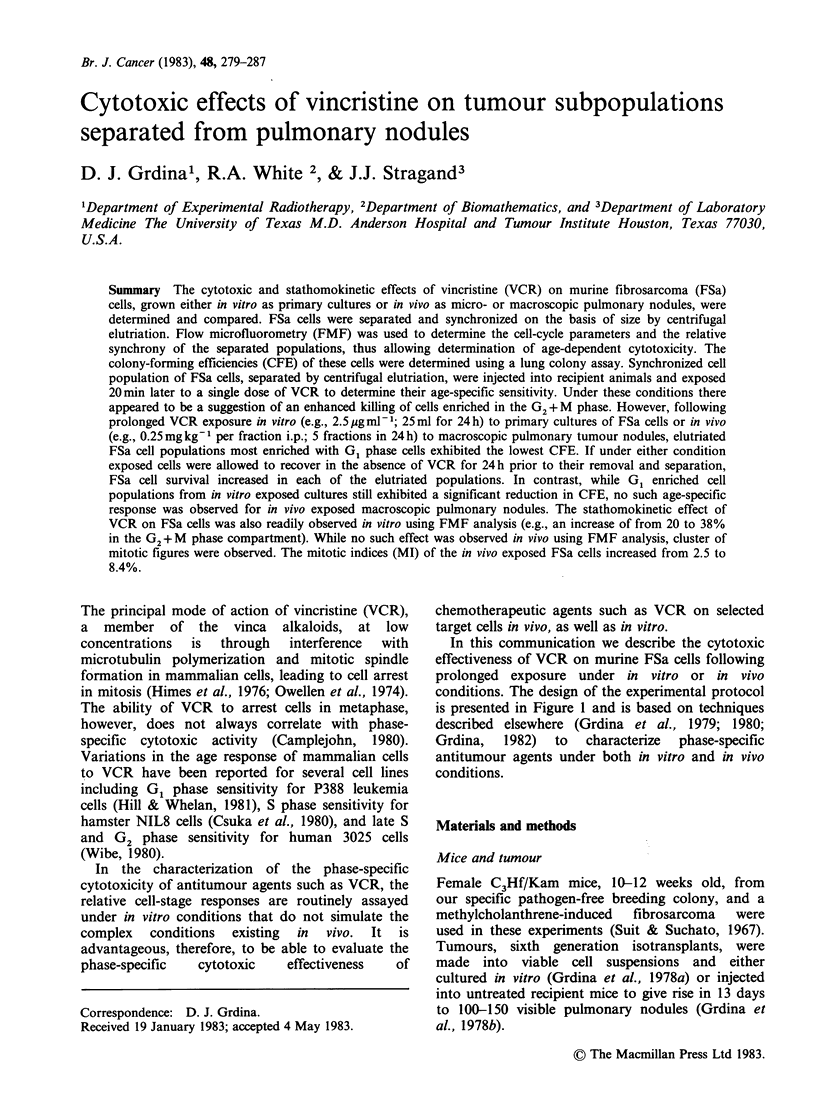

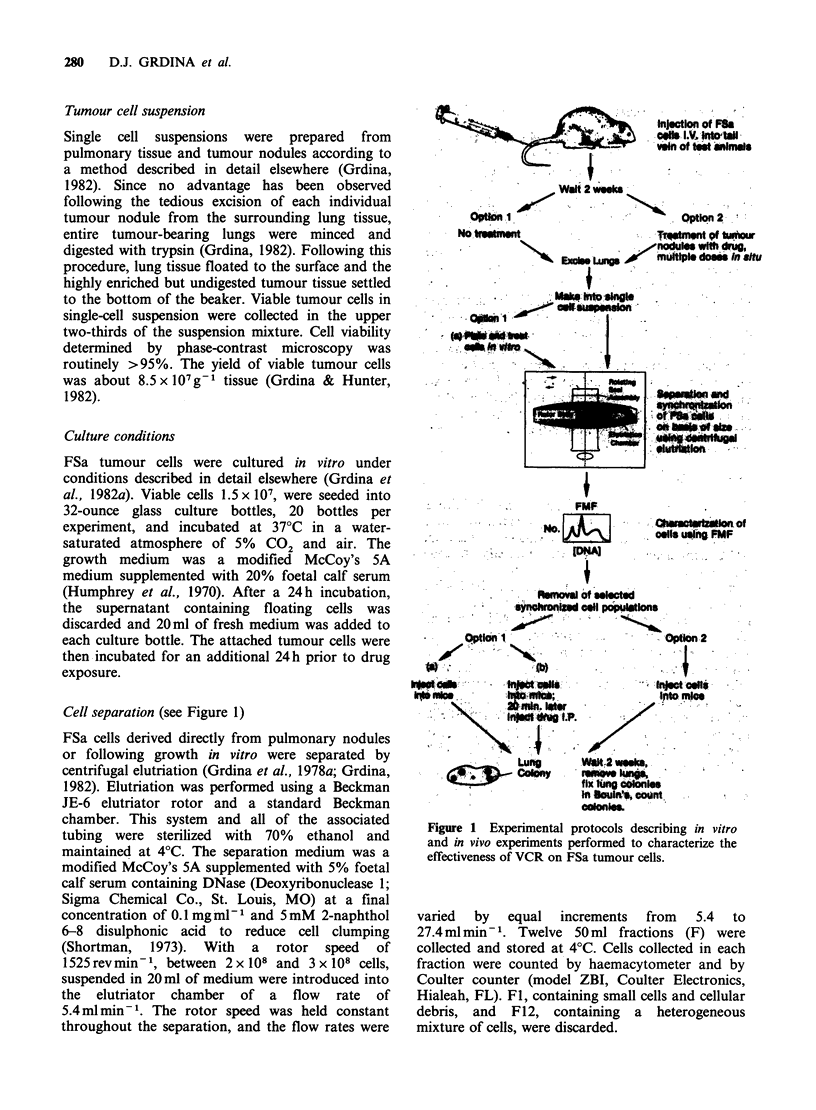

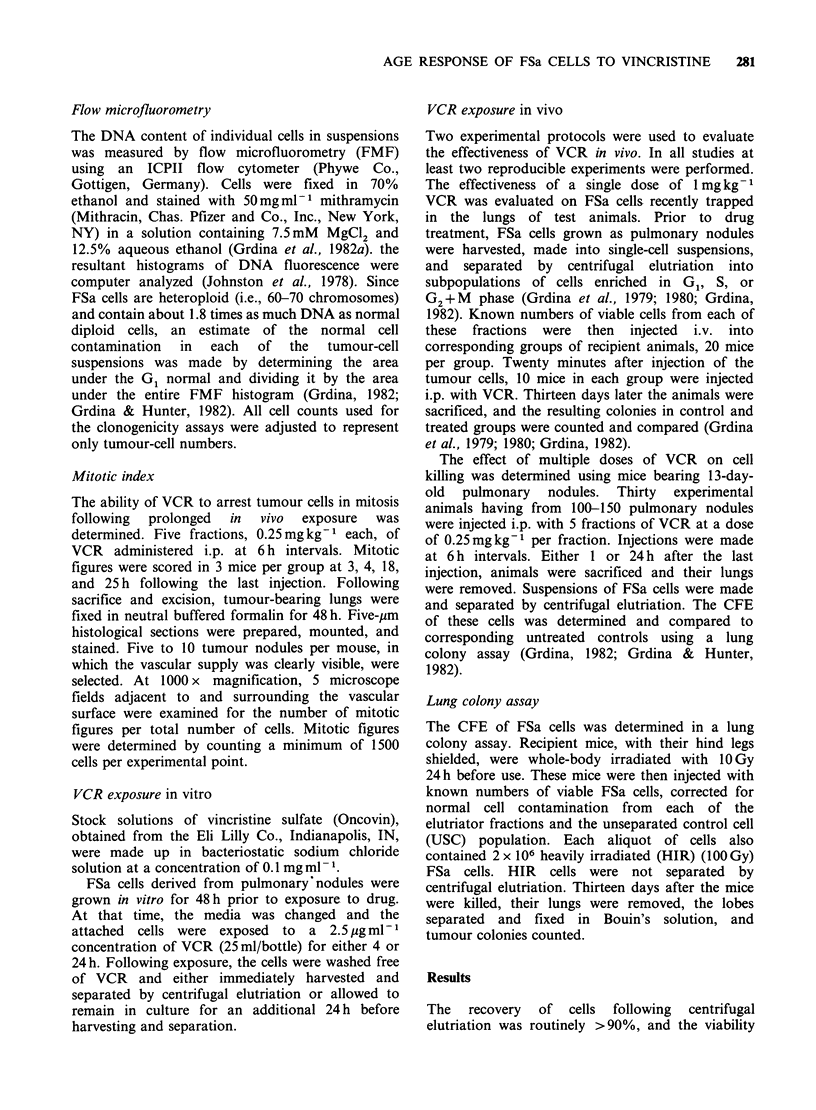

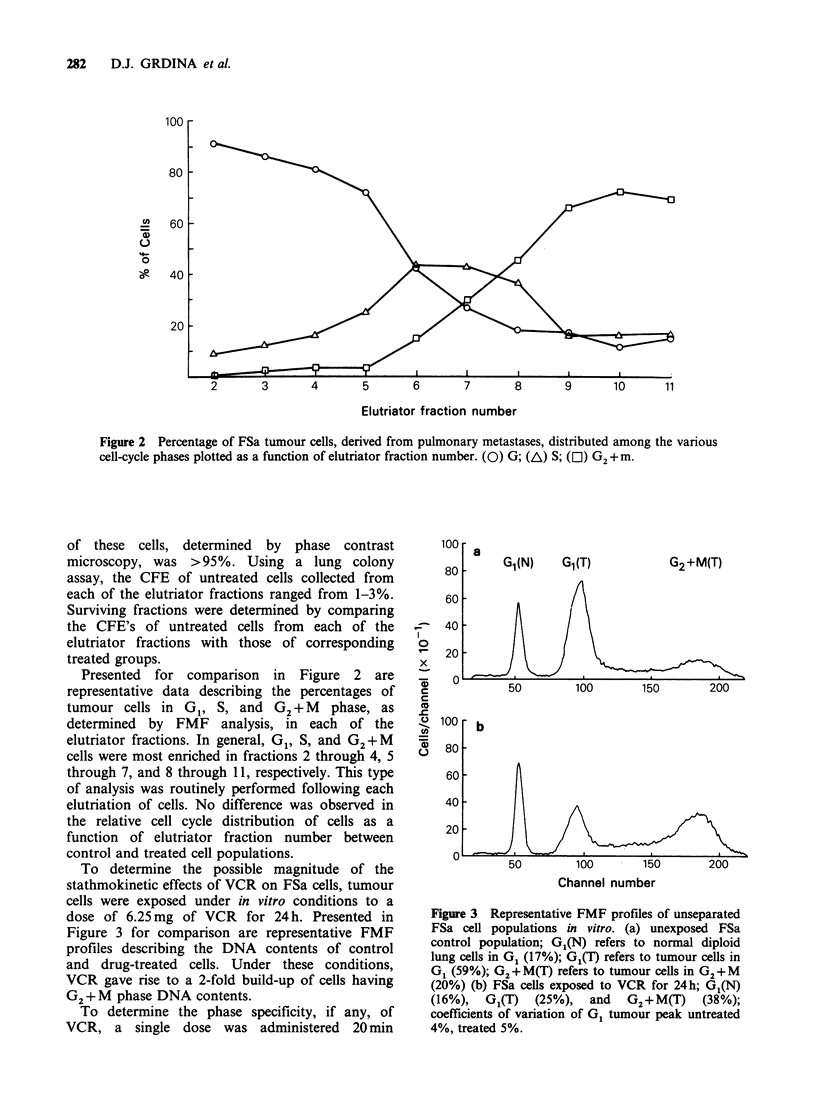

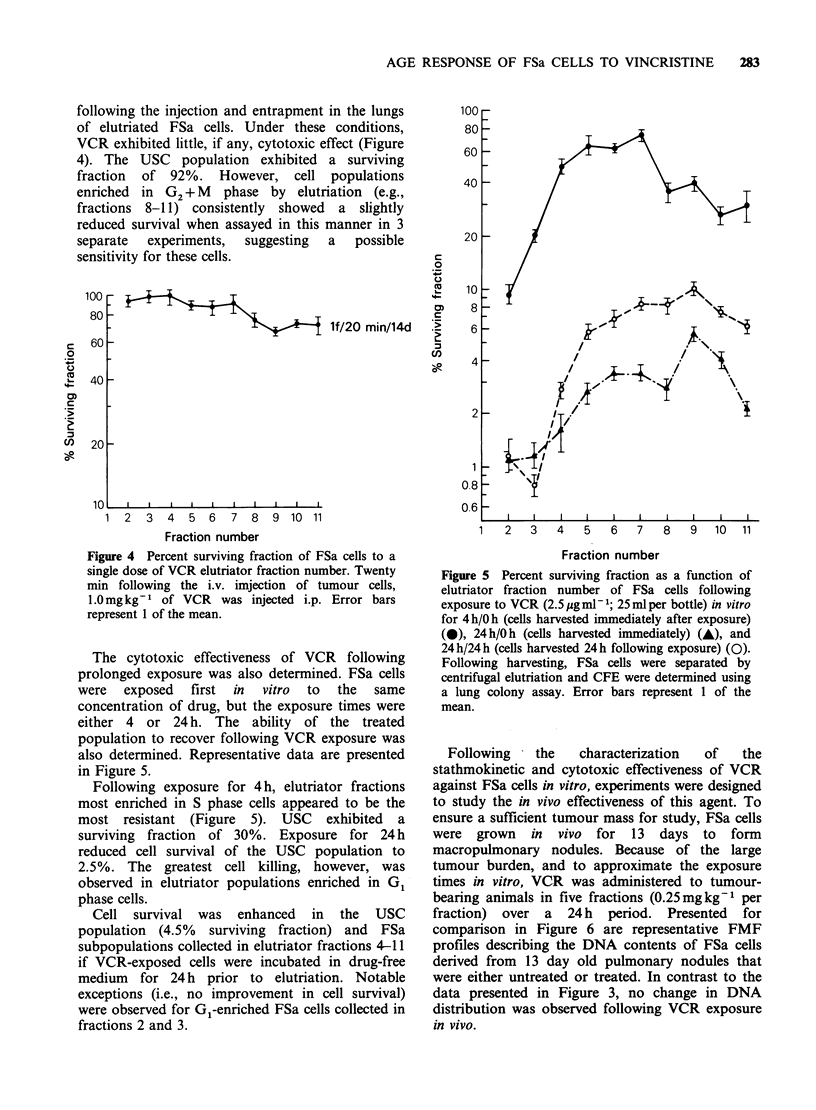

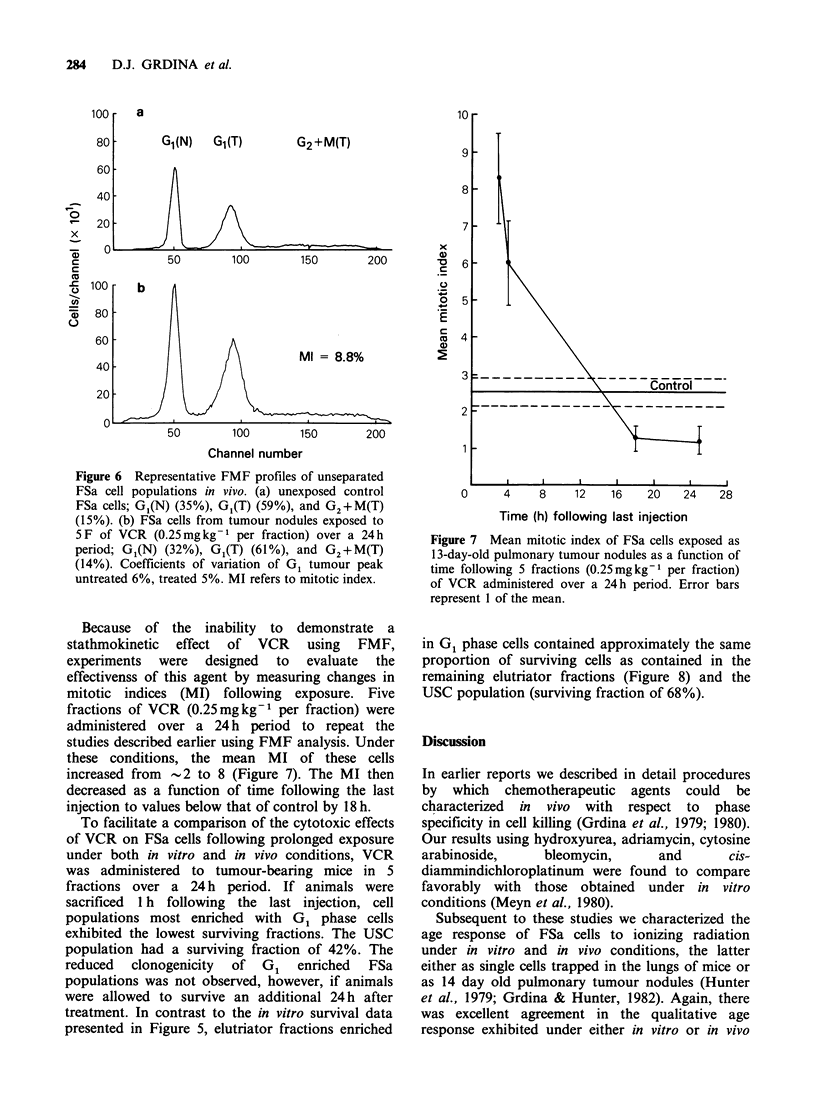

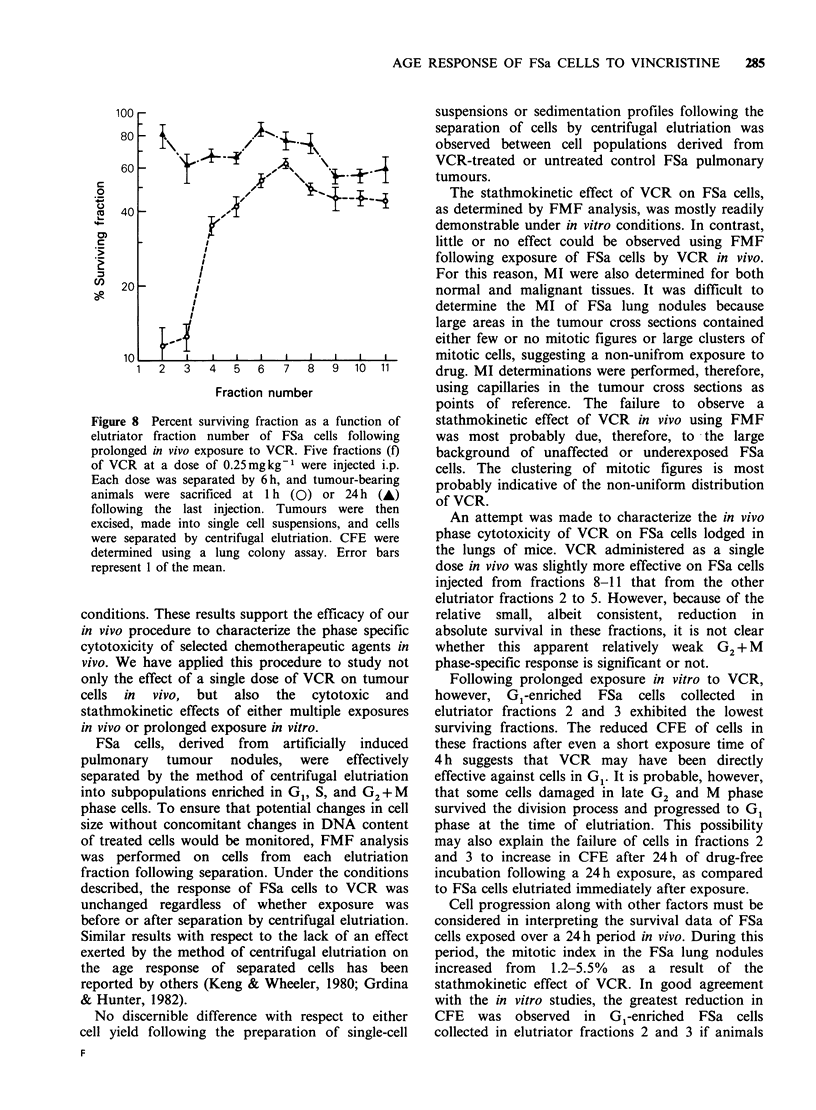

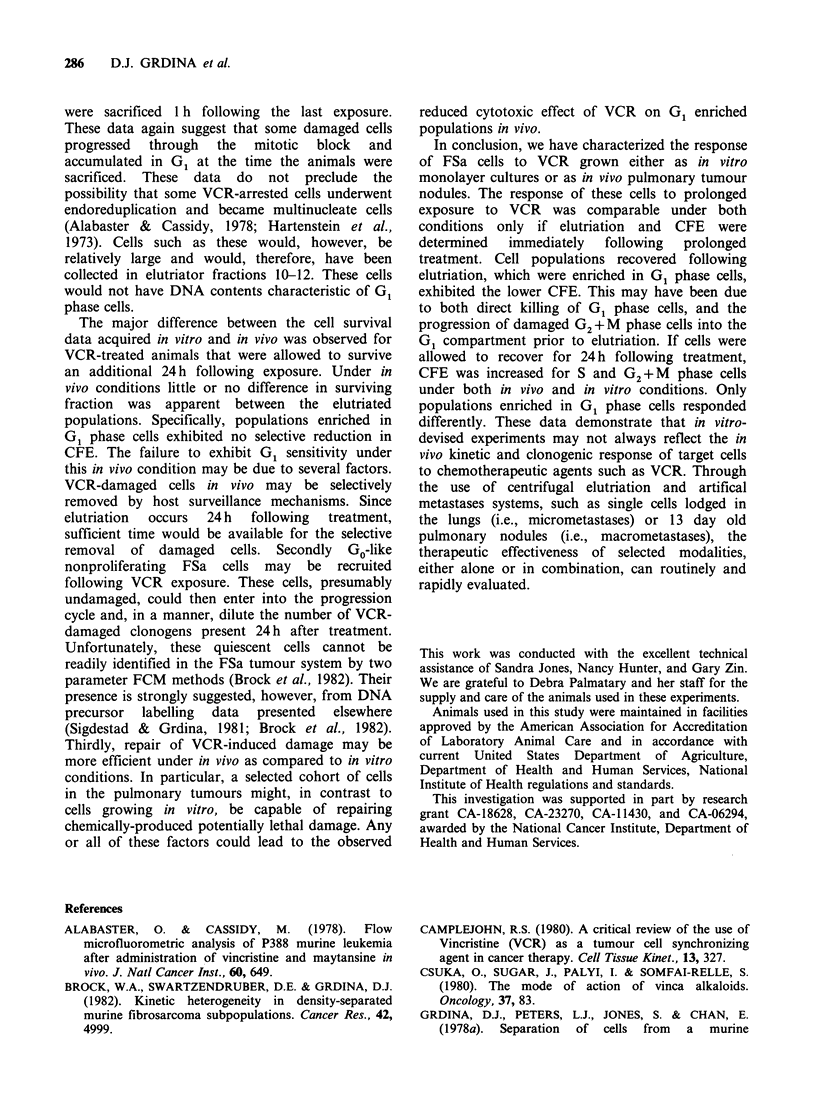

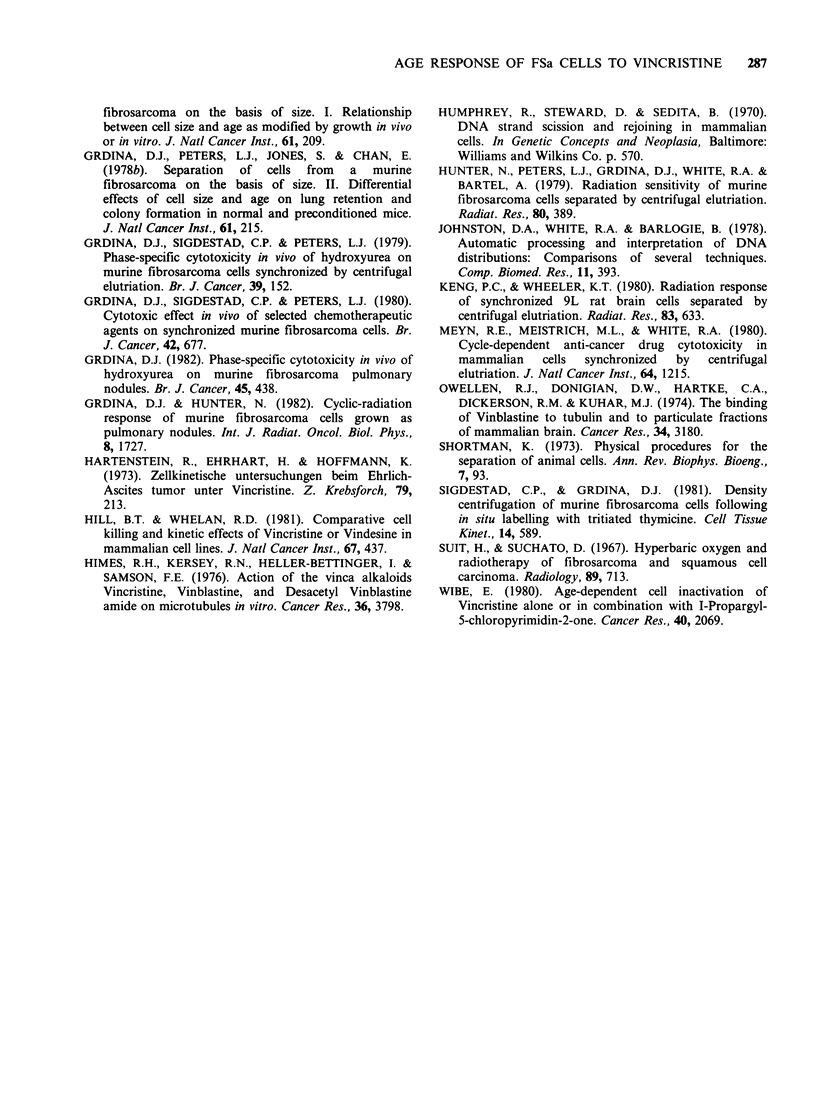


## References

[OCR_00917] Alabaster O., Cassidy M. (1978). Flow microfluorometric analysis of P388 murine leukemia after administration of vincristine and maytansine in vivo.. J Natl Cancer Inst.

[OCR_00923] Brock W. A., Swartzendruber D. E., Grdina D. J. (1982). Kinetic heterogeneity in density-separated murine fibrosarcoma subpopulations.. Cancer Res.

[OCR_00929] Camplejohn R. S. (1980). A critical review of the use of vincristine (VCR) as a tumour cell synchronizing agent in cancer therapy.. Cell Tissue Kinet.

[OCR_00934] Csuka O., Sugár J., Pályi I., Somfai-Relle S. (1980). The mode of action of Vinca alkaloids.. Oncology.

[OCR_00974] Grdina D. J., Hunter N. (1982). Cyclic-radiation response of murine fibrosarcoma cells grown as pulmonary nodules.. Int J Radiat Oncol Biol Phys.

[OCR_00939] Grdina D. J., Peters L. J., Jones S., Chan E. (1978). Separation of cells from a murine fibrosarcoma on the basis of size. I. Relationship between cell size and age as modified by growth in vivo or in vitro.. J Natl Cancer Inst.

[OCR_00949] Grdina D. J., Peters L. J., Jones S., Chan E. (1978). Separation of cells from a murine fibrosarcoma on the basis of size. II. Differential effects of cell size and age on lung retention and colony formation in normal and preconditioned mice.. J Natl Cancer Inst.

[OCR_00969] Grdina D. J. (1982). Phase-specific cytotoxicity in vivo of hydroxyurea on murine fibrosarcoma pulmonary nodules.. Br J Cancer.

[OCR_00963] Grdina D. J., Sigdestad C. P., Peters L. J. (1980). Cytotoxic effect in vivo of selected chemotherapeutic agents on synchronized murine fibrosarcoma cells.. Br J Cancer.

[OCR_00957] Grdina D. J., Sigdestad C. P., Peters L. J. (1979). Phase-specific cytotoxicity in vivo of hydroxyurea on murine fibrosarcoma cells synchronized by centrifugal elutriation.. Br J Cancer.

[OCR_00980] Hartenstein R., Ehrhart H., Hoffmann K. (1973). Zellkinetische Untersuchungen beim Ehrlich-Ascites-Tumor unter Vincristin.. Z Krebsforsch Klin Onkol Cancer Res Clin Oncol.

[OCR_00986] Hill B. T., Whelan R. D. (1981). Comparative cell killing and kinetic effects of vincristine or vindesine in mammalian cell lines.. J Natl Cancer Inst.

[OCR_00991] Himes R. H., Kersey R. N., Heller-Bettinger I., Samson F. E. (1976). Action of the vinca alkaloids vincristine, vinblastine, and desacetyl vinblastine amide on microtubules in vitro.. Cancer Res.

[OCR_01003] Hunter N., Peters L. J., Grdina D. J., White R. A., Bartel A. (1979). Radiation sensitivity of murine fibrosarcoma cells separated by centrifugal elutriation.. Radiat Res.

[OCR_01009] Johnston D. A., White R. A., Barlogie B. (1978). Automatic processing and interpretation of DNA distributions: comparison of several techniques.. Comput Biomed Res.

[OCR_01015] Keng P. C., Wheeler K. T. (1980). Radiation response of synchronized 9L rat brain tumor cells separated by centrifugal elutriation.. Radiat Res.

[OCR_01020] Meyn R. E., Meistrich M. L., White R. A. (1980). Cycle-dependent anticancer drug cytotoxicity in mammalian cells synchronized by centrifugal elutriation.. J Natl Cancer Inst.

[OCR_01026] Owellen R. J., Donigian D. W., Hartke C. A., Dickerson R. M., Kuhar M. J. (1974). The binding of vinblastine to tubulin and to particulate fractions of mammalian brain.. Cancer Res.

[OCR_01037] Sigdestad C. P., Grdina D. J. (1981). Density centrifugation of murine fibrosarcoma cells following in situ labelling with tritiated thymidine.. Cell Tissue Kinet.

[OCR_01043] Suit H. D., Suchato C. (1967). Hyperbaric oxygen and radiotherapy of a fibrosarcoma and of a squamous-cell carcinoma of C3H mice.. Radiology.

[OCR_01048] Wibe E. (1980). Age-dependent cell inactivation by vincristine alone or in combination with 1-propargyl-5-chloropyrimidin-2-one.. Cancer Res.

